# Effect of roof colour on indoor temperature and human comfort levels, with implications for malaria control: a pilot study using experimental houses in rural Gambia

**DOI:** 10.1186/s12936-021-03951-4

**Published:** 2021-10-29

**Authors:** Majo Carrasco-Tenezaca, Ebrima Jatta, Musa Jawara, John Bradley, Margaret Pinder, Umberto D’Alessandro, Jakob Knudsen, Steve W. Lindsay

**Affiliations:** 1 Department of Biosciences, Durham University, Durham, UK; 2 National Malaria Control Programme, Banjul, The Gambia; 3 Medical Research Council Unit The Gambia at the London School of Hygiene & Tropical Medicine, Banjul, The Gambia; 4 London School of Hygiene & Tropical Medicine, MRC International Statistics and Epidemiology Group, London, UK; 5 Department of Disease Control, London School of Hygiene & Tropical Medicine, London, UK; 6 Schools of Architecture, Design and Conservation, The Royal Danish Academy of Fine Arts, Copenhagen, Denmark

**Keywords:** Malaria, Sub-Saharan Africa, Housing, Roofs, Indoor temperature, Human comfort

## Abstract

**Background:**

In rural sub-Saharan Africa, thatch roofs are being replaced by metal roofs. Metal roofing, however, increases indoor temperatures above human comfort levels, and thus makes it more likely that residents will not use an insecticide-treated bed net (ITN) at night. Whether the colour of a metal roof affects indoor temperature and human comfort was assessed.

**Methods:**

Two identical, experimental houses were constructed with metal roofs in rural Gambia. Roof types were: (1) original bare-metal, (2) painted with red oxide primer or (3) white gloss, to reflect solar radiation. Pairwise comparisons were run in six, five-night blocks during the malaria season 2018. Indoor climate was measured in each house and multivariate analysis used to compare indoor temperatures during the day and night.

**Results:**

From 21.00 to 23.59 h, when most residents decide whether to use an ITN or not, the indoor temperature of a house with a bare metal roof was 31.5 °C (95% CI  31.2–31.8 °C), a red roof, 30.3 °C (95% CI 30.0–30.6) and a white roof, 29.8 °C (95% CI 29.4–30.1). During the same period, red-roofed houses were 1.23 °C cooler (95% CI 1.22–1.23) and white roofs 1.74 °C cooler (95% CI 1.70–1.79) than bare-metal roofed houses (*p*  < 0.001). Similar results were found from 00.00 to 06.00 h. Maximum daily temperatures were 0.93 °C lower in a white-roofed house (95% CI  0.10–0.30, *p*  < 0.001), but not a red roof (mean maximum temperature difference  = 0.44 °C warmer, 95% CI  0.43–0.45, *p*  = 0.081), compared with the bare-metal roofed houses. Human comfort analysis showed that from 21.00 to 23.59 h houses with white roofs (comfortable for 87% time) were more comfortable than bare-metal roofed houses (comfortable for 13% time; odds ratio  = 43.7, 95% CI 27.5–69.5, *p * < 0.001). The cost of painting a metal roof white is approximately 31–68 USD.

**Conclusions:**

Houses with a white roof were consistently cooler and more comfortable than those with a bare metal roof. Painting the roofs of houses white is a cheap way of making a dwelling more comfortable for the occupants and could potentially increase bed net use in hot humid countries.

**Supplementary Information:**

The online version contains supplementary material available at 10.1186/s12936-021-03951-4.

## Background

Between 2000 and 2015, the percentage of improved houses, classified as those with improved water and sanitation, sufficient living area and constructed from durable materials, increased from 8.2 to 18.4% in rural sub-Saharan Africa and from 32.3 to 53.2% in urban areas [1]. At the same time, the region is experiencing an unprecedented increase in population and it is estimated that by 2050, there will be an extra one billion people living in the region [2], mainly in informal settlements [3]. This rise in population will be accompanied by a huge demand for new and improved housing.

Many of these new homes will be built in malaria-endemic areas and will require novel methods of disease control. Currently, one of the principal tools for malaria control are insecticide-treated bed nets (ITNs) [4], which provide protection against malaria vectors feeding indoors at night. Correct use of ITNs can be a highly effective intervention [4]. In many countries, however, especially when it is hot and humid, nets are considered too hot to sleep under [5]. Simple solutions for cooling down houses at night are thus required to increase net usage and reduce malaria prevalence.

One of the most obvious and rapid transformations in housing seen in sub-Saharan Africa is that metal roofs are replacing traditional thatched roofs [1] (Additional File 1: Figure S1). Studies in the hot, humid tropics have shown that metal-roofed houses are hotter during the day than those that have thatched roofs and they remain appreciably hotter well into the night [6–8]. Thus whilst, high indoor temperatures experienced in metal-roofed houses during the day increases the mortality of malaria mosquitoes resting indoors [9], thereby reducing transmission, they also increase the risk that people at night will not sleep under a net [5] due the remaining heat emitted mainly by walls.

**Fig. 1:**
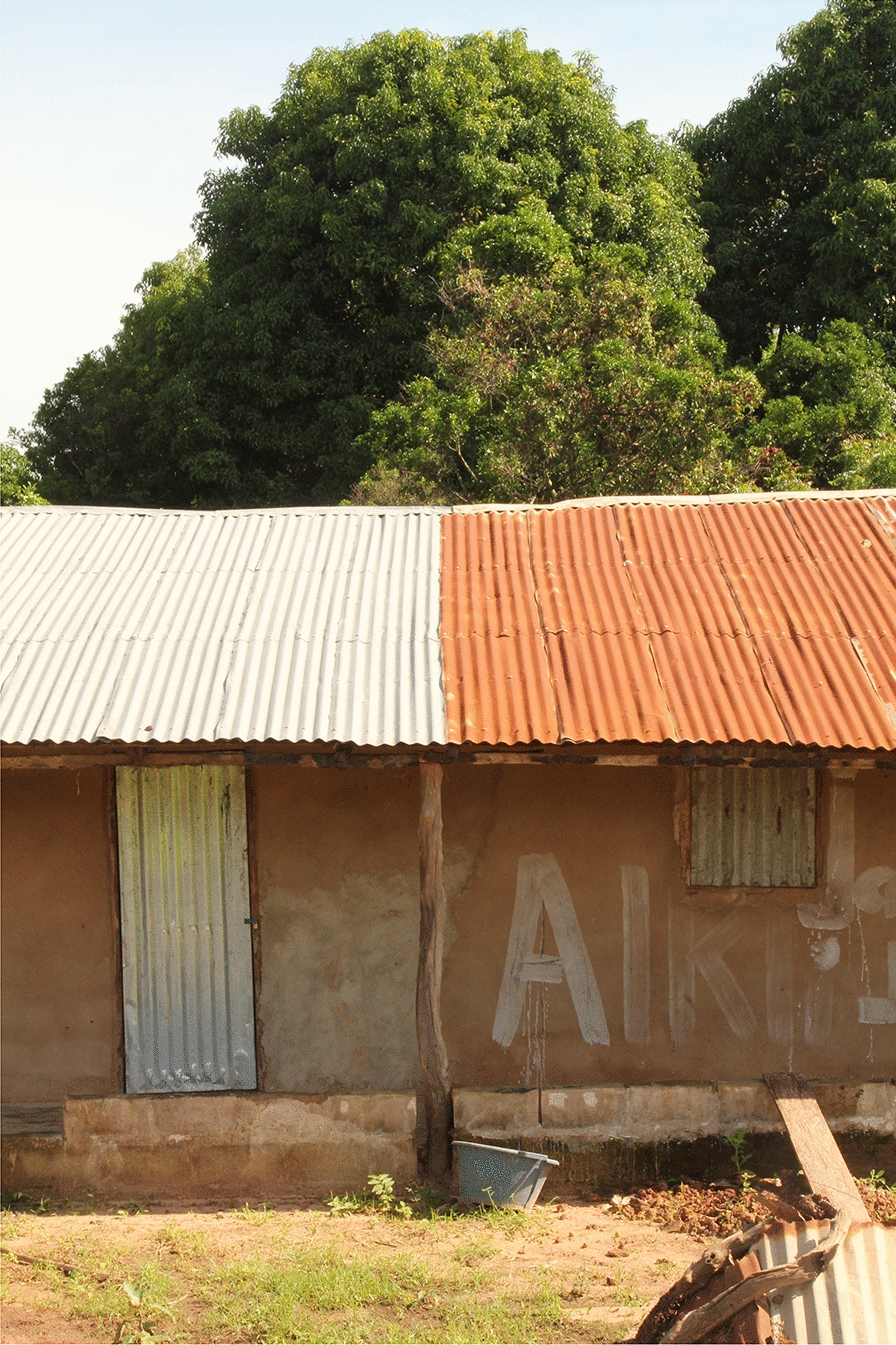
A new (left) and rusted metal roof (right) in a Gambian village

Typically, houses in sub-Saharan Africa have a roof built from a single layer of thin steel corrugate sheeting, coated with zinc for increased durability and protection against corrosion. Roofs protect the main structure of the house and in harsh environments, like that experienced by much of the region, are exposed to high levels of solar radiation, temperature, rain and wind. Over time, metal corrodes due to oxidation caused by contact with water [10], salt, dust and soot [11, 12], causing the roof to rust. This process of oxidation changes the colour of the roof from silvery grey to dark red (Fig. 1) and the surface becomes rugous, both factors that affect the physical characteristics of the roof. To slow oxidation, further prolong the useful life of zinc-treated sheeting, and enhance the appearance of these houses, pre-painted corrugate sheets of different colours can be purchased at a higher cost price, alternatively the zinc-coated roofing is frequently painted, after purchase by householders.

Roofs transmit the heat they absorb from solar radiation to the indoor space they cover, therefore in hot environments, their characteristics are crucial to keep the house cool. Having a light-coloured or white roof can reduce indoor temperatures. In Arizona, USA, surface temperatures of white roofs were 20 °C–30 °C lower than those of grey or brown roofs as a result of the greater reflectance of white roofs [13]. High solar reflectance will reduce the heat transmitted to the indoor space. In India, painting metal roofs with solar reflective paint reduced indoor air temperatures by − 5 °C at the hottest time of the day, compared to untreated houses [14].

In this study, the impact of different colour of metal roof on the indoor temperatures of basic housing in a tropical setting was examined. In general, light colours absorb less heat from the sun than darker colours. Thus, the study tested two hypotheses: (1) that a house with a white roof would be cooler than one with an unpainted metal roof, and (2) a house with a red roof would be hotter than one with an unpainted roof. These hypotheses were tested using experimental houses in rural Gambia.

## Methods

### Study design

**Fig. 2:**
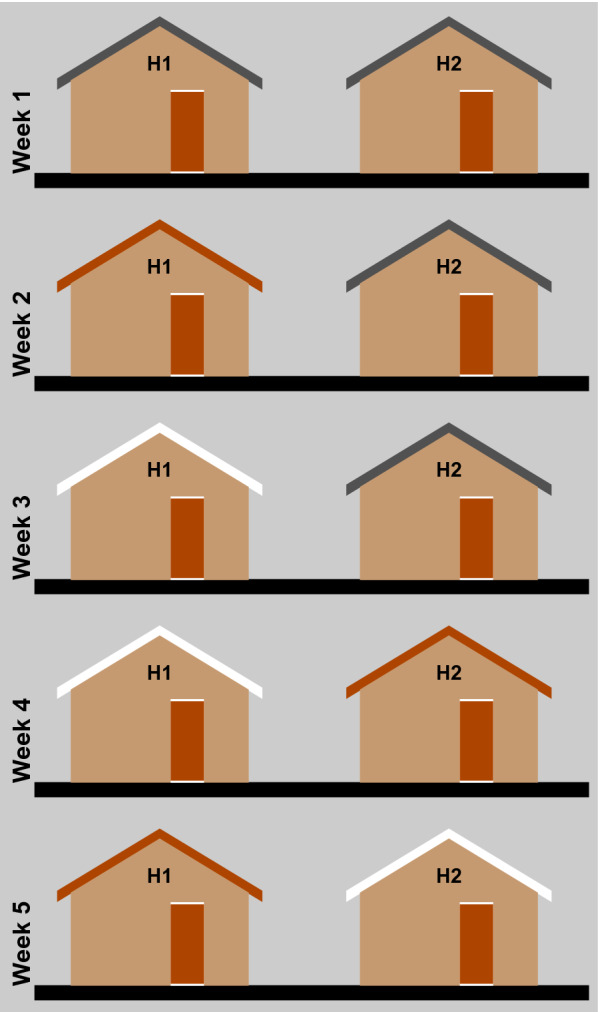
Experimental schedule. Where, bare metal roofs are shown in grey, red roofs in red and white roofs in white. H1 and H2 are the house numbers

This was an experimental study using two identical uninhabited houses with metal saddle-shaped roofs constructed from corrugated, galvanized metal sheeting carried out in the field. Three types of roof were tested: (1) bare metal, (2) a roof painted with red oxide primer and (3) one painted with white gloss paint. After the baseline week, when both houses had unpainted metal roofs, in weeks 2, 3 and 4, one roof was painted each week with either red or white paint and in week 5 the colours were switched (Fig. 2). Indoor and outdoor temperature and relative humidity were monitored continuously during the study. The primary objective was to determine whether indoor temperature and thus, human comfort, changed according to the colour of the metal roof. The houses had two bamboo beds with mattresses but no inhabitants.

### Study area

The study took place at the Medical Research Councils Unit The Gambia’s, field station at Wali Kunda (N 13° 34∙25′, W 14° 55∙28′), located on the south bank of the River Gambia in the Central River Region, The Gambia. The study took place during the rainy season, from September 11th to October 21st 2017, when numbers of the malaria vector, *Anopheles gambiae *sensu lato (s.l.), are greatest [8].

### Experimental houses

**Fig. 3:**
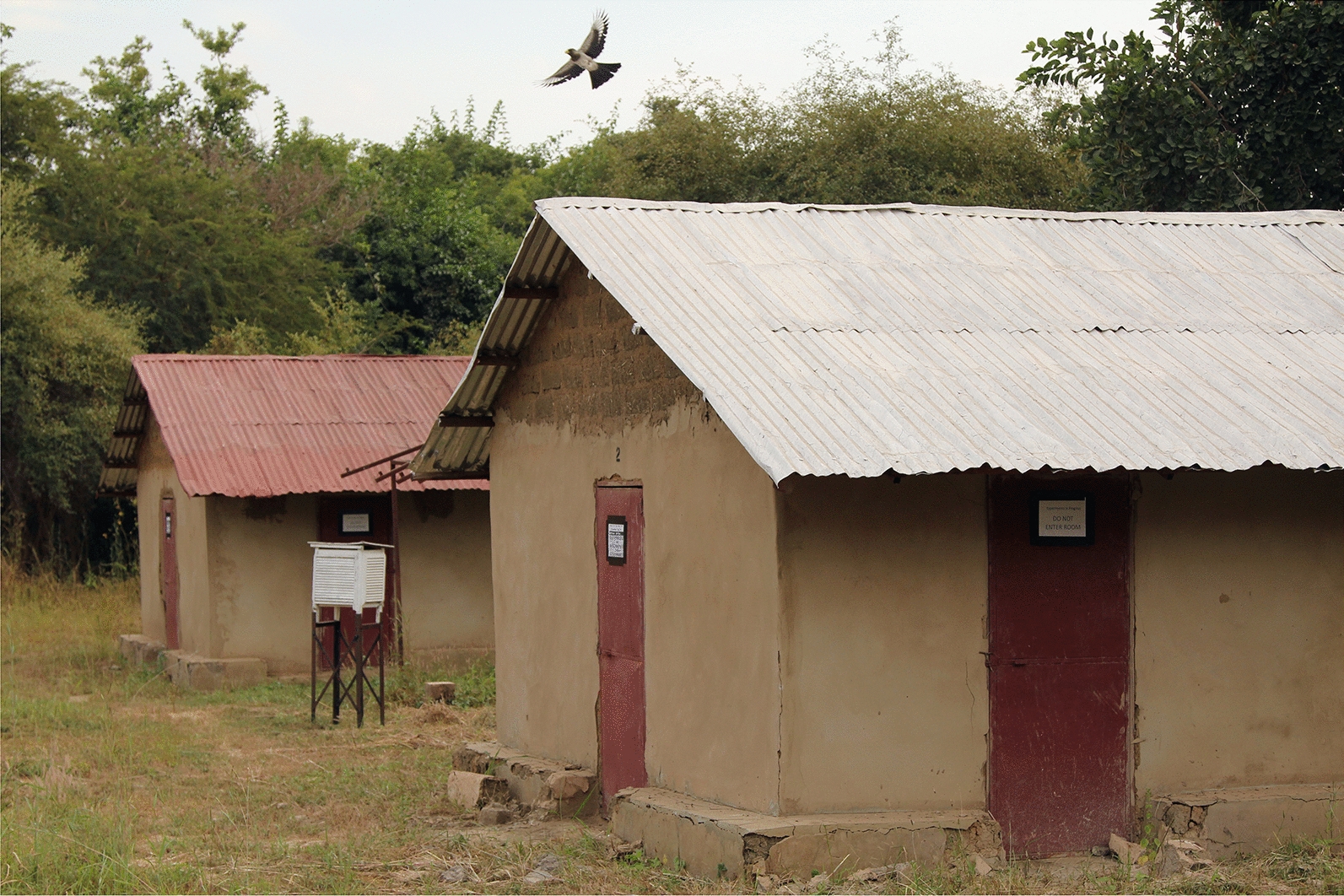
Position of experimental houses and outdoor weather station

Construction of the experimental houses have been described in detail elsewhere [15]. Two experimental houses were the same size as the average one-roomed rural house, 10 m apart (Fig. 3). Each house was constructed from mud blocks, covered with a thin mud and cement render and had a galvanized corrugated roof with closed eaves (the gap between the top of the wall and the roof), as is typical of metal-roofed housing in The Gambia. The dimensions of the roof were 4.80 m by 2.42 m and the corrugate metal was 0.14 mm thick. Each house was 4.20 m by 4.20 m in floor area and the walls were 2.20 m high. Both houses had three doors located on three different walls, and each door was 1.80 m high and 0.80 m wide. There were no windows in the houses, as is common in many rural homes.

### Experiments

Five, one weekly experiments were conducted, each one of five night’s duration. In the baseline week, both houses had the same bare metal roofs to simulate typical metal-roofed houses found in local villages and to determine whether there were inherent differences in temperature between the two houses. Thereafter, each week one or both roofs was painted a different colour, first either red or white, using the schedule shown in Fig. 2. Bare-metal roofs were first painted with red matt paint (Red Oxide paint, National Paints Factories, Dubai), used as a primer for metal surfaces, and then painted with one coat of white gloss (White Oil paint, National Paints Factories, Dubai). The roof colour was changed weekly in a progressive fashion to avoid having to return to bare-metal roofs. It also required a roof to be painted first with primer and then gloss paint to reduce the number of paint coats on the roof that could affect the heating and cooling of the roof. To change a white roof to a red roof, it was simply painted over the white paint with one coat of red primer. The first house to have a metal roof painted was randomly selected by flipping a coin.

### Environmental measurements

Indoor temperature and relative humidity were measured in each house every 30 min using data loggers (TGU 4500, Tinytag, UK) positioned in the centre of the room, 1 m above the floor. Outdoor temperature and relative humidity were recorded every 30 min with a data logger inside a Stevenson screen located mid-way between the two houses.

### Cost estimation

The cost of installing and, where appropriate, painting a gable roof for an average single-room rural Gambian house was estimated. The cheapest roofing option available in the market is corrugated galvanized steel which comes in a variety of sizes and thicknesses, with 1.85 m by 0.65 m being the most common. The experimental houses had 28 smaller metal sheets, each 0.65 m by 1.85 m, to cover the 23.23 m^2^ roof area. To paint a red roof requires one coat of red oxide paint primer, whilst a white roof requires one coat of red oxide primer and two coats of white gloss over the primer. Each coat of paint needed to cover an area of 23.23 m^2^ requires 2.90 L of paint, assuming a coverage of 8 m^2^ L^−1^. (Coverage was taken from manufacturer technical specifications at www.hammerite.com, accessed on February 8th, 2021, Hammerite, Northumberland, UK). Thus, to paint a roof white, a householder would need 5 L of red oxide primer for the first coat and 10 L of white gloss paint for two additional coats. Paint costs would be further reduced by painting more than one house at a time, preventing waste of paint. An alternative would be to use pre-painted coloured metal sheets, which have a high albedo [16]. Costs of labour, painting equipment and shipment were considered the same for each roof type so were not included in the analysis. The costs of materials from The Gambia and South Africa were used to explore ranges in prices. Costs are presented in USD based on an exchange rate of 1 Gambian dalasi  =  0.019 USD and 1 South African Rand  =  0.061 USD (Morningstar at Google accessed 19th October 2020).

### Data analysis

The two primary outcomes were mean indoor temperature and human comfort in the first part of the night, when people choose whether to use a bed net or not. Differences between outside and indoor temperatures were used to make comparisons between houses with different coloured roofs. Linear regression was used to assess the effect of roof colour on indoor temperature, for indoor temperature and relative humidity, since both variables were continuous and normally distributed. In addition to roof colour, house position, coats of paint and collection night number were included in the model as fixed effects. Temperature was analysed in three periods: (1) 21.00–23.59 h, going to bed, (2) 00.00–6.59 h, sleeping, and (3) 07.00–20.59 h, awake, according to observations made in a local Gambian village [17].

For the analysis of indoor temperature between houses IBM SPSS Statistics version 20 and Stata version 16 was used. This experiment was a pilot exercise, designed to inform future studies about sample sizes. The LadyBug (LadyBug Products, Athol, ID, USA) software package was used to estimate the proportion of time putative occupants would spend in the so-called ‘comfort zone’ which is determined by indoor levels of temperature, relative humidity, indoor wind strength and the activity and clothing of the inhabitants. The human comfort index is widely used by those working in the built environment to assess how comfortable a building is and is based on experiments with hundreds of volunteers of different genders, ages and ethnicity wearing different amounts of clothing [18]. The index is used in the ANSI/ASHRAE Standard 55: Thermal Environmental Conditions for Human Occupancy. It is an American National Standard published by ASHRAE that establishes the ranges of indoor environmental conditions to achieve acceptable thermal comfort for occupants of buildings [19]. Readings that fall in the comfort polygon provide an estimated proportion of time that people would be comfortable, not too hot nor too cold. The model was parameterised assuming that from 21.00 to 23.59 h two adults in each houses would be sitting chatting and wearing thin straight trousers and briefs, and from 00.00 to 06.59 h people inside the houses would be sleeping without sheeting and the indoor air speed was 0 ms^−1^. For each house typology, the proportion of time the indoor climate was within the comfort zone was calculated for both periods: when people retire to bed and when they are sleeping. Comparisons of human comfort were made using chi-square tests using Epiinfo (version 7).

## Results

**Fig. 4:**
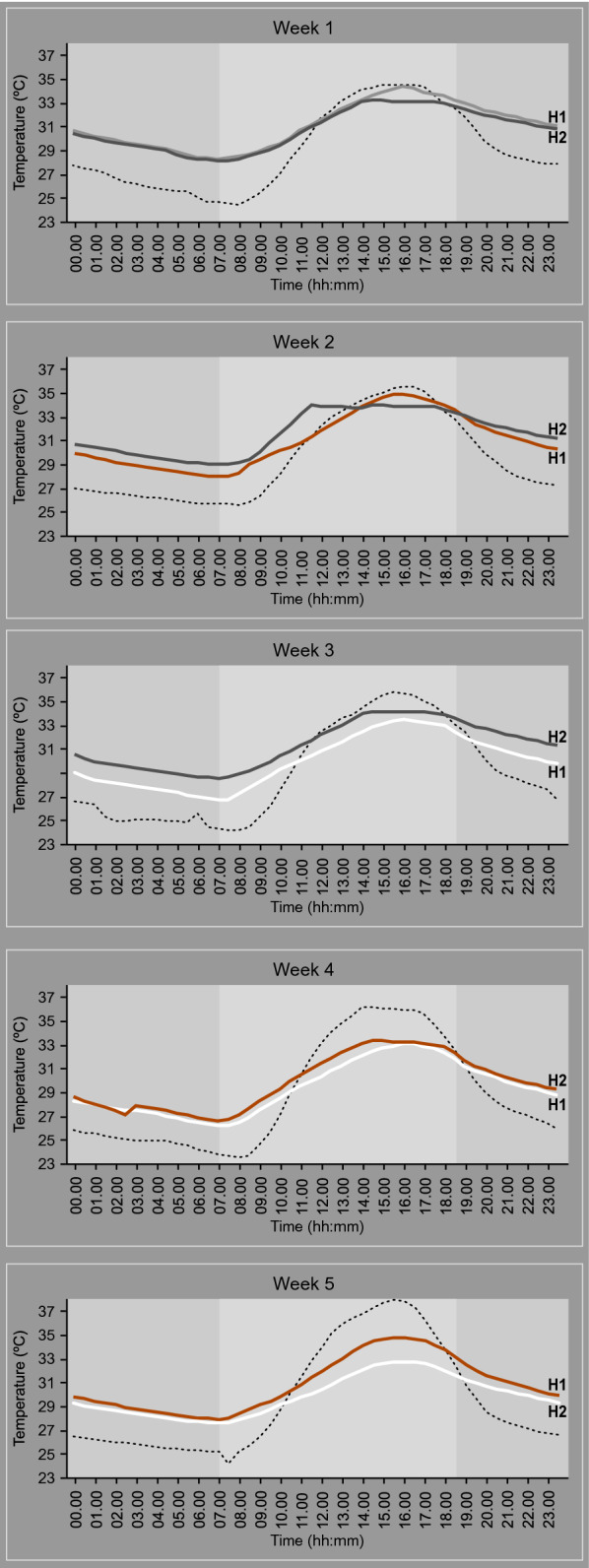
Mean hourly temperature for each roof typology. Where bare metal roofs are shown in black or grey, red roofs in red and white roofs in white. Dotted line is outdoor temperature and light grey bar marks sunrise to sunset

Both houses showed a similar pattern of daily temperature cycles (Fig. 4). At night, it was hotter indoors than outdoors, whilst during the late afternoon the temperatures were similar or hotter outdoors. Indoor temperatures warmed during the day, reaching maximum temperatures between 14.00 and 18.00 h, before declining slowly during the night. In week one, when both houses had bare metal roofs, house 1 had a maximum temperature of 35.6 °C at 16.00 h and a minimum temperature 26.5 °C at 06.00 h. In contrast, house 2, had a maximum temperature of 34.5 °C at 14:30 h and the minimum at 06.00 h with 25.8 °C. The maximum temperature in house 1 was more pronounced than house 2, which appeared flattened, probably due to the shade of tall trees that fell across the roof of house 2 in the late afternoon. Outdoor temperatures and relative humidity experienced during the study is shown in the supplementary material (Additional File 1: Figure S1).

During the study, white roofs were consistently cooler throughout the day compared with the other roof types (Table 1). Red roofs were generally cooler than the metal roofs, except during the hottest part of the day between 15.00 and 16.00 h.

**Table 1:** Indoor and outdoor temperatures at night and during the day

	Type of roof	Mean indoor temperature ( °C)	Temp. difference ( °C)	Temp. difference ( °C)	p value
			Indoors vs. outdoors	From bare metal-roofed house	
Night—1st part	Bare metal	31.49 (31.19–31.79)	3.37 (2.13–3.60)	–	–
(21.00–00.00 h)	Red	30.26 (29.96–30.57)	3.08 (2.80–3.36)	− 1.23 (− 1.22 to − 1.23)	< 0.001
	White	29.75 (29.40–30.09)	2.51 (2.25–2.76)	− 1.74 (− 1.70 to − 1.79)	< 0.001
Night—2nd part	Bare metal	29.43 (29.13–29.73)	3.41 (3.12–3.70)	–	–
(00.30–07.00 h)	Red	28.42 (28.05–28.79)	2.78 (2.63–2.93)	− 1.01 (− 0.94 to − 1.08)	< 0.001
	White	27.84 (27.43–28.25)	2.49 (2.26–2.72)	− 1.59 (− 1.48 to − 1.70)	< 0.001
Day	Bare metal	33.98 (33.52–34.44)	− 1.41 (− 1.71 to – 1.12)	–	–
(07:30–20.30 h)	Red	34.42 (33.95–34.89)	− 2.47 (− 3.13 to − 1.82)	0.44 (0.43–0.45)	0.081
	White	33.05 (32.70–33.40)	− 3.93 (− 4.75 to 3.11)	− 0.93 (− 0.82 to − 1.04)	< 0.001
Daily	Bare metal	30.69 (30.31–31.06)	2.10 (1.63–2.58)	–	–
(00:00–23:30 h)	Red	29.91 (29.48–30.33)	1.37 (0.97–1.77)	− 0.63 (− 0.44 to − 0.82)	0.001
	White	29.15 (28.71–29.58)	0.82 (0.21–1.43)	− 1.44 (− 1.41 to − 1.46)	< 0.001

Adjusted analysis for night, house position and number of coats of paint

Figures in parentheses are 95% confidence intervals

Data calculated with mean values for “night” and “daily”, and maximum values for “day” recorded and 95% CIs. General linearized modelling results adjusted for house position, roof typology, layers of paint and night

Both during the day and night, multivariate analysis adjusting for house position, number of paint coats and night number, showed that a house with a white roof was cooler than one with a bare-metal roof (Table 1). Red roof houses were also cooler than those with bare metal roofs during the night, but not during the day, and were not as cool as white roofed houses. There was no association between indoor temperatures and number of coats of paint on the roofs.

**Fig. 5:**
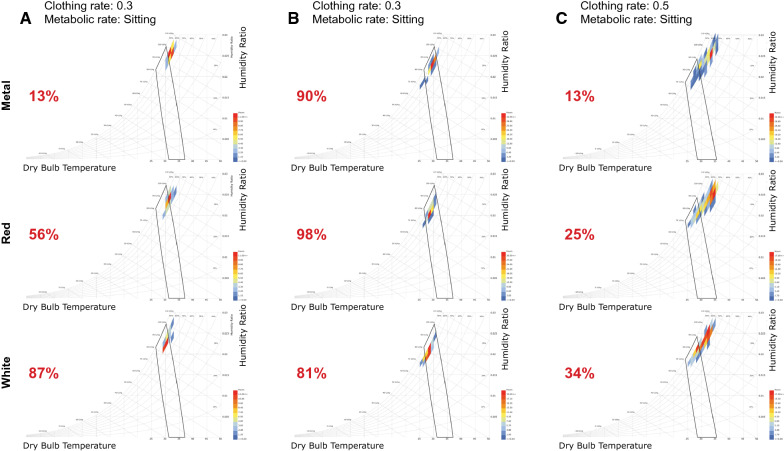
Psychrometric charts showing the human comfort index of adults. Data modelled for houses with metal, red and white roofs during three different intervals of the day. Where **A** night first part (21:00–23:30 h), **B **night second part (00:00–07:00 h) and **C **day time (07.00–20:30 h). Each data point represents a combination of dry-bulb temperature and relative humidity recorded by indoor loggers. Recordings made within the black polygons represent values that are comfortable. Values in red are the percentage of readings that are classified as comfortable

The percentage of time each roof typology was comfortable varied at different times of the day and night (Fig. 5). Before midnight, it was more comfortable in red-roofed houses (56% of the time, Odds ratio, OR  =  8.44, 95% CI 5.67–12.57, p  < 0.001) and white-roofed houses (87% time, OR  =  43.69, 95% CI 27.46–69.52, p  < 0.001), than bare metal-roofed houses (13% time). After midnight, all houses were comfortable over 80% of the time, however, red-roofed houses (98% of the time, OR, R  =  5.63, 95% CI  2.31–13.69, p  < 0.001) were more comfortable, and white-roofed houses (81% time, OR  = 0.47, 95% CI 0.03–0.75, p  < 0.001) less comfortable, because they were colder, than bare metal-roofed houses (90% time). During the day, all of the houses were comfortable less than 35% of the time. It was more comfortable inside red-roofed houses (25% of the time, OR  = 2.20, 95% CI 1.45–3.34, p  < 0.001) and white-roofed houses (34% time, OR  = 3.40, 95% CI 2.27–5.09, p  < 0.001) than bare metal-roofed houses (13% time). Overall, white-roofed houses were the most comfortable.

### Roof costings

**Fig. 6:**
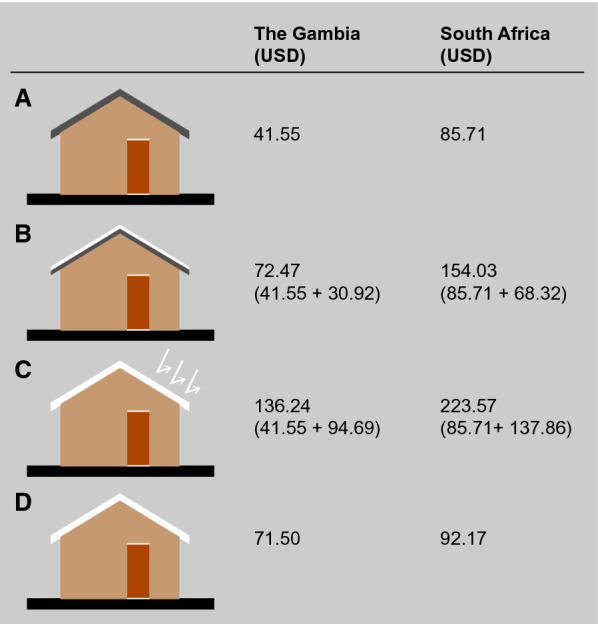
Roof costing with different technical specifications. Where **A **galvanized corrugated roof; **B **galvanized corrugated roof with white coating; **C **galvanized corrugated roof with white reflective coating; **D **fabric-coloured roof. All prices were converted from Gambian Dalasis and South African Rands to USD, by Morningstar at Google on 19th October 2020

The cost of a white roof varies according to the quality of paint and which country one buys materials in (Fig. 6). The cost of installing a metal roof in The Gambia, assuming the wooden roof frame was in place and there were no local transport costs would be 41.55 USD. A white-painted roof would cost 72.47 USD, including the price of metal sheets, and a roof coated with paint designed to be highly reflective would cost 136.24 USD. A factory-coloured metal roof would cost 71.50 USD. Prices will vary widely across sub-Saharan Africa, according to the quality of the paint and the country of purchase. For example, 5 L of white gloss paint can vary in price between 6.76 USD and 13.53 USD in The Gambia and 15.13 USD and 48.68 USD in South Africa (prices accessed and converted to USD on 19th October 2020). In the Upper River Region, the median number of residents in a single room house is four (Q1  = 1, Q3  = 5, n  = 3253 data collected from 13 Nov 2014 to 25 Mar 2015; M. Pinder, pers. commun.). Assuming this, a painted white-roof last 10 years, the cost per individual each year would be 1.81 USD.

## Discussion

A white metal roof painted made the house cooler during the day and night compared with an unpainted roof. Houses with white-gloss roofs were cooler than unpainted metal roofs at all times of day: 1.7 °C cooler from 21.00 to 23.59 h, 1.6 °C cooler from 00.00 to 05.59 h, and 0.9 °C cooler from 07:00 to 20:30 h, the hottest time of the day. A matt-red roof, simulating a rusty roof, was cooler at night, before midnight, than a house with an unpainted metal roof, but was as hot as a unpainted metal roof during the hottest time of the day, between 14.00 and 17.00 h. Houses with red-matt roofs were 1.2 °C cooler from 21.00 to 23.59 h, 0.95 °C cooler from 00.00 to 05.59 h, compared with unpainted metal-roofed houses. Although red-roofed houses were 0.44 °C warmer than unpainted metal roofs during the hottest time of the day, this was not statistically significant.

A number of other studies have also found that white-roofed houses in the tropics are cooler than other roof colours [14, 20, 21]. Most of these studies, however, measured surface temperature of the roof or compared indoor temperature in houses with traditional and modern roofs. In Argentina, white-painted concrete roofs recorded average surface temperatures of 43.0 °C, while terracotta-painted ones were 59.5 °C [11]. Three years later, when the roofs were re-surveyed, surface temperatures were 47.5 °C for white roofs and 63.5 °C for terracotta roofs, suggesting that white paint did not deteriorate over this period and continued to be cooler compared with terracotta roofs. The white-roof coating also prevented deterioration of the roofing because it has higher solar reflectance and, therefore, reduced thermal expansion and contraction that causes cracks [21]. In the Dominican Republic, during the day, galvanized corrugated roofs painted white had surface temperatures of 33 °C, whilst those painted dark red paint had temperature of 42 °C [22]. In this experiment having a white-painted roof white increased the reflectance of light from 70 to 80% and probably increased the life of a roof by preventing rusting. In Ecuador, a study with 23 roofing samples commonly used for government-funded housing concluded an increase in the hours of comfort could be achieved by using higher solar reflectance roofing [16], findings consistent with our own study.

The analysis suggested that the number of paint layers applied to a roof did not affect indoor temperature. Galvanized metal roof sheets sold in The Gambia are thin, only 0.11–0.45 mm thick, and a layer of paint adds little insulation, therefore, heat from solar radiation does not accumulate in the metal and is transferred rapidly to the surrounding air [22]. This aligns with the literature that describes an insulating material as one that has high thermal mass to reduce the maximum daytime temperature [21].

Indoor temperature decreased during the night following a similar pattern to that observed in previous studies [8, 12]. Indoor temperatures were consistently warmer at night than outdoor temperatures. During the first and second parts of the night, on average unpainted metal roofed houses were 3.37 °C and 3.41 °C warmer, red-roofed houses 3.08 °C and 2.78 °C warmer, and white-roofed houses 2.51 °C and 2.49 °C warmer than outdoor temperatures. In marked contrast, during the day, the maximum indoor temperature in the unpainted metal-roof houses was 1.41 °C cooler, red-roofed houses 2.47 °C cooler and white-roofed houses was 3.93 °C cooler than outdoors in the shade.

White-roofed houses were the least uncomfortable during the day, but were still too uncomfortable for most of the day, with indoor temperatures regularly exceeding 30 °C. Nonetheless, before midnight they are the most comfortable houses with 87% of the evening being comfortable for people. Since this is the period when children [23] and adults [17] go to sleep, they may be more likely to use an ITN than those sleeping in houses with unpainted or red/rusted roofs. After midnight, people in white-roofed houses are slightly less comfortable than those in red-roofed houses as they would start to feel cold. Feeling cold though is easily solved by using a bedsheet, unlike being too hot which can only be reduced by increasing ventilation or sleeping with a damp towel. Lower indoor temperatures in poorly ventilated white-roofed houses will also reduce the concentration of carbon dioxide produced by people [24]. This is important since this gas is a major mosquito attractant, thus white-roofed houses may attract fewer malaria mosquitoes than houses with galvanized metal roofs. Painting a metal roof white is relatively cheap and would cost 41.55 USD and could last 5–10 years. As a malaria intervention it would cost 1.04–2.08 USD per person, which is favourable compared to the cost of ITNs (2.20 USD per person) and indoor residual spraying (6.70 USD per person) [25].

There are several limitations to this study. Firstly, the study was conducted in two experimental houses, thus only two different roof typologies could be tested simultaneously. Secondly, the study was conducted in single-room houses, which is not representative of all ethnic groups in The Gambia. In Mandinka villages, for example, most houses are multiple-roomed and built along a line [12], larger roof areas could increase heat accumulation or disperse it. Also, the increase in buildings in the surroundings could prevent air flowing close to the house, cooling it down. Thirdly, the roof was painted with dark red paint to simulate the colour of a rusted roof. The change in roof colour, however, may not mimic the surface changes caused by rusting. Thus, a rough rusted roof may be hotter than the smoother red-painted roofs. Nonetheless, many houses in The Gambia are painted with red oxide paint, to prevent rusting and so the red roof is representative of a roof type common in the country. Fourthly, the experimental houses were not inhabited at night, which would have raised indoor temperatures at night.

The increase in use of corrugate roofs throughout sub-Saharan Africa, reflects the need for cheap, durable, readily available materials for quick and simple construction. In contrast, traditional thatched roofs, although cheaper, are difficult to install well and need replacing every 2–3 years. Painting metal roofs requires specialist paints and could cost up to four times the price of normal wall paints, but results in a surface that will not peal over time and can be applied on rusty surfaces. Today, corrugate roof sheets are available in different colours in the market at the same price as non-coated ones (Additional file 2: Figure S2), but at almost twice the cost of galvanized metal sheets, which are the most popular roof choice in sub-Saharan Africa. The principal driver of the rainbow of different roof colours is one of satisfying personal choice, rather than being motivated by an effort to improve comfort levels indoors. To reduce indoor temperature and increase reflectance, roofs should be covered with reflective paint. The cost of which can double the cost of normal metal paint. Light-coloured roofs are likely to have high acceptance among local rural populations because corrugate roofs are perceived as a symbol of modernity and wealth. Further research is needed to assess acceptance of the colour, especially since light-coloured materials show dirt more readily than darker colours, and the possible association of white with a mourning colour in the region. More importantly, studies need to address whether lower indoor temperatures will increase ITN usage and reduce malaria transmission.

## Conclusions

The findings show that the colour of a roof impacts on indoor temperature in rural houses. Compared to a conventional, unpainted galvanized metal roof, a white-painted roof decreases indoor temperature both during the day and night. Importantly, in tropical areas a white-painted roof might make it more likely that residents would sleep under a bed net at night and protect themselves from vector-borne diseases. A white-painted roof may last up to 10 years, increase the life span of the metal roof and, at a cost of 1.04 USD per person per year, is relatively cheap. Further cooling indoors could be achieved by installing screened windows on opposite walls [24]. Painting rural roofs with white paint will help make a dwelling more comfortable for the occupants and potentially increase bed net use in hot humid countries.

## Supplementary Information

**Additional file 1: Figure S1.** Outdoor temperature and humidity during the study. Grey = bare metal roofs, red = red roof and white = white roof.

**Additional file 2: Figure S2. **Aerial view showing different roof colours in Tanzania © Jakob B. Knudsen.

## Data Availability

The datasets used and/or analysed during this study are available from the corresponding author on reasonable request.

## Abbreviations

ITNs: Insecticide-treated nets
USD: United States dollar
