# Supplementary figures and images for: Effect of roof colour on indoor temperature and human comfort levels, with implications for malaria control: a pilot study using experimental houses in rural Gambia
Source: Malar J. 2021 Oct 29;20:423. doi: 10.1186/s12936-021-03951-4 (PMC8555212; doi:10.1186/s12936-021-03951-4)

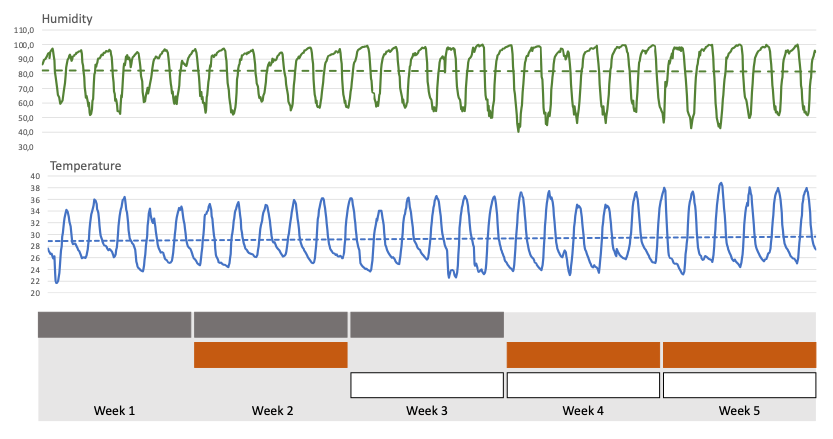

Supplement: Supplementary file 1 — Additional file 1: Figure S1. Outdoor temperature and humidity during the study. Grey = bare metal roofs, red = red roof and white = white roof. [file 12936_2021_3951_MOESM1_ESM.png]

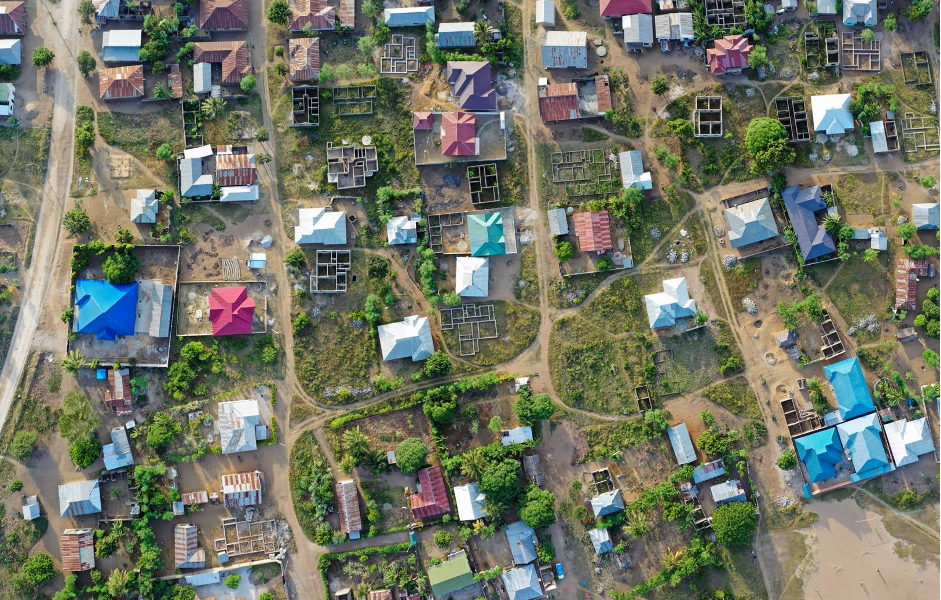

Supplement: Supplementary file 2 — Additional file 2: Figure S2. Aerial view showing different roof colours in Tanzania © Jakob B. Knudsen. [file 12936_2021_3951_MOESM2_ESM.png]
